# ATM, ATR and DNA-PKcs expressions correlate to adverse clinical outcomes in epithelial ovarian cancers

**DOI:** 10.1016/j.bbacli.2014.08.001

**Published:** 2014-08-14

**Authors:** Tarek M.A. Abdel-Fatah, Arvind Arora, Paul Moseley, Clare Coveney, Christina Perry, Kerstie Johnson, Christopher Kent, Graham Ball, Stephen Chan, Srinivasan Madhusudan

**Affiliations:** a Department of Oncology, Nottingham University Hospitals, Nottingham NG5 1PB, UK; b Laboratory of Molecular Oncology, Division of Oncology, School of Medicine, University of Nottingham, Nottingham University Hospitals, Nottingham NG5 1PB, UK; c School of Science and Technology, Nottingham Trent University, Clifton Campus, Nottingham NG11 8NS, UK

**Keywords:** ATM, ATR, DNA-PK, Ovarian cancer, Biomarker

## Abstract

**Background:**

Ataxia-telangiectasia mutated (ATM), ataxia-telangiectasia mutated and rad3 related (ATR) and DNA-dependent protein kinase catalytic sub-unit (DNA-PKcs) play critical roles in DNA damage response (DDR) by linking DNA damage sensing to DDR effectors that regulate cell cycle progression and DNA repair. Our objective was to evaluate if ATM, ATR and DNA-PKcs expressions could predict response to therapy and clinical outcome in epithelial ovarian cancers.

**Methods:**

We investigated ATM, ATR, and DNA-PKcs expressions in ovarian epithelial cancers [protein expression (n = 194 patients), mRNA expression (n = 156 patients)] and correlated to clinicopathological outcomes as well as expression of X-ray repair cross-complementing protein 1 (XRCC1), cell division cycle-45 (CDC45), cyclin-dependent kinase 1(CDK1) and Ki-67 in tumours.

**Results:**

High ATM protein expression was associated with serous cystadenocarcinomas (p = 0.021) and platinum resistance (p = 0.017). High DNA-PKcs protein expression was associated with serous cystadenocarcinomas (p = 0.006) and advanced stage tumours (p = 0.018). High ATM protein (p = 0.001), high ATM mRNA (p = 0.018), high DNA-PKcs protein (p = 0.002), high DNA-PKcs mRNA (p = 0.044) and high ATR protein (p = 0.001) expressions are correlated with poor ovarian cancer specific survival (OCSS). In multivariate Cox model, high DNA-PKcs (p = 0.006) and high ATR (p = 0.043) protein expressions remain independently associated with poor OCSS.

**Conclusions:**

ATM, ATR and DNA-PKcs expressions may have prognostic and predictive significances in epithelial ovarian cancer.

**General significance:**

The data presented here provides evidence that ATM, ATR and DNA-PKcs involved in DDR are not only promising biomarkers but are also rational targets for personalized therapy in ovarian cancer.

## Highlights

• ATM, ATR and DNA-PKcs are key factors involved in DNA damage response (DDR).
• We investigated ATM, ATR and DNA-PKcs in ovarian cancer.
• High levels of ATM, ATR and DNA-PKcs have prognostic significance.
• High level of ATM predicts resistance to cisplatin therapy.

## 1 Introduction

Despite the efficacy of platinum based chemotherapy, the overall prognosis for patients with advanced ovarian cancer remains poor [1], [2], [3]. Resistance to platinating agents (carboplatin, cisplatin) is a formidable clinical problem and may be directly related to proficient DNA damage signalling and DNA repair in cancer cells [4], [5]. ATM (ataxia-telangiectasia mutated), ATR (ataxia-telangiectasia mutated and Rad3 related) kinases and DNA-PKcs (DNA-dependent protein kinase catalytic sub-unit) play critical roles in the DNA damage response (DDR) and link DNA damage sensing to DDR effectors that regulate cell cycle progression and DNA repair [6], [7], [8], [9], [10], [11], [12], [13], [14]. Whereas ATM and DNA-PK are predominantly activated by DNA double strand breaks (DSBs) [6], [7], [8], [9], [12], ATR is activated in response to a number of DNA damaging lesions that involve single-stranded (SS)–double-stranded (DS) junctions such as those generated when replication fork encounters a DNA lesion or during nucleotide excision repair or during resection of a DSB [10], [11], [13], [14]. Activated ATR and ATM phosphorylate Chk1 or Chk2 respectively. This in turn modulates a number of other proteins involved in DNA repair, cell cycle control and apoptosis [6], [7], [8], [9], [10], [11], [12], [13], [14]. Significant crosstalk and redundancy also occur between the ATR, ATM and DNA-PKcs pathways in order to maintain genomic stability in cells [15], [16], [17], [18].

Given the complex network and the critical role in DDR we hypothesized and have provided evidence here that ATR, ATM and DNA-PKcs expressions have prognostic and predictive significances in ovarian cancer patients.

## 2 Methods

### 2.1 Protein expression cohort

Investigation of the expression of ATR, ATM, DNA-PKcs, XRCC1, Ki-67, CDC45 and CDK1, in ovarian epithelial cancer was carried out on tissue microarrays of 195 consecutive ovarian epithelial cancer cases treated at Nottingham University Hospitals (NUH) between 2000 and 2007. Patients were comprehensively staged as per the International Federation of Obstetricians and Gynaecologists (FIGO) Staging System for Ovarian Cancer. Survival was calculated from the operation date until the 1st of October 2012 when any remaining survivors were censored. Patient demographics are summarized in Supplementary Table 1. Platinum resistance was defined as patients who had progression during first-line platinum chemotherapy or relapse within 6 months after completion of platinum treatment.

### 2.2 Tissue microarray (TMA) and immunohistochemistry (IHC)

TMAs were constructed as described previously [19]. Briefly, triplicate tissue cores with a diameter of 0.6 mm were taken from the tumour and arrayed into a recipient paraffin block using a tissue puncher/arrayer (Beecher Instruments, Silver Spring, MD, USA) as previously described [19]. Four micron sections of the tissue array block were cut and placed on Surgipath X-tra Adhesive microscope slides (Leica Microsystems) for immunohistochemical staining.

Immunohistochemical staining for ATR, ATM, DNA-PK, XRCC1 Ki-67, CDC45 and CDK1 was performed using Thermo Scientific Shandon Sequenza chambers and the Leica Novolink max polymer detection system (RE7280-K) according to manufacturer instructions (Leica Microsystems). Pre-treatment of TMA sections was performed with either citrate buffer (pH 6.0, 20 min, Microwave) or EDTA (pH 8.0, 25 min, hot water bath), depending on the antibody (Supplementary Table S2). Sections heated in EDTA required a chamber suspended in a hot water bath, followed by gradual cooling-transfer to warm TBS solution for 10 min prior to immersion in cold water. TMA sections were incubated at room temperature with each antibody according to optimal conditions and summarized in supplementary Table S2. Optimization protocols for ATM, ATR and DNA-PKcs are summarized in Supplementary data 1. Negative controls with no primary antibody were included in each run and shown in Supplementary Fig. S1.

### 2.3 Evaluation of immune staining

The tumour cores were evaluated by expert pathologists blinded to the clinico-pathological characteristics of patients in two different settings. There was excellent intra and inter-observer agreements (*k* > 0.8; Cohen's *κ* and multi-rater *κ* tests, respectively). Whole field inspection of the core was scored, the sub cellular localisation of each marker was identified (nuclear, cytoplasm, cell membrane), and the optimal scoring methodology was applied in each case (summarized in Supplementary Table S2). Intensities of subcellular compartments were each assessed and grouped as follows: 0 = no staining, 1 = weak staining, 2 = moderate staining, 3 = strong staining. The percentage of tumour cells in each category was estimated (0–100%). H-score (range 0–300) was calculated by multiplying the intensity of staining and the percentage of staining. Not all cores within the TMA were suitable for IHC analysis due to missing cores or absence of tumour cells.

### 2.4 Gene expression cohort

We performed gene expression studies in an ovarian cancer cohort consisting of 156 patients treated at the University Medical Centre, Groningen, Netherlands. The original study describing the demographics and treatment characteristics has been published by Crijns et al. [20]. Briefly, median age was 60 years (range 21–84), 79.6% of patients had FIGO stage IIIC disease, and all patients were treated according to the Dutch guidelines and received cytoreductive surgery followed by platinum based chemotherapy. Tumour samples were microarray profiled on the Operon v3.0 probes two colour oligonucleotide microarrays [21]. The microarray data are accessible at the National Center for Biotechnology Information (NCBI) Gene Expression Omnibus (http://www.ncbi.nlm.nih.gov/geo/ via series accession number GSE13876) and can also be downloaded from the Array express data set E-GEOD-13876 (http://www.ebi.ac.uk/arrayexpress/experiments/E-GEOD-13876/). Data from the gene probes relating to ATM, ATR and DNA-PK were extracted and a survival analysis was performed on the expression values. All data were normalized using the global mean method (MAS5), and probe set signal intensities were natural log transformed and scaled by adjusting the mean signal to a target value of log 500.

### 2.5 Statistical analysis

Data analysis was performed using SPSS (SPSS, version 17 Chicago, IL). Where appropriate, Pearson's Chi-square, Fisher's exact, Student's t and ANOVA one way tests were used. Cumulative survival probabilities were estimated using the Kaplan–Meier method, and differences between survival rates were tested for significance using the log-rank test. Multivariate analysis for survival was performed using the Cox hazard model. The proportional hazard assumption was tested using standard log–log plots. Hazard ratios (HR) and 95% confidence intervals (95% CI) were estimated for each variable. p Value < 0.05 was considered significant.

The Reporting Recommendations for Tumor Marker Prognostic Studies (REMARK) criteria, recommended by McShane et al. [22], were followed throughout this study. This work was approved by the Nottingham Research Ethics Committee.

## 3 Results

### 3.1 Clinicopathological correlations

#### 3.1.1 ATM

**Fig. 1:**
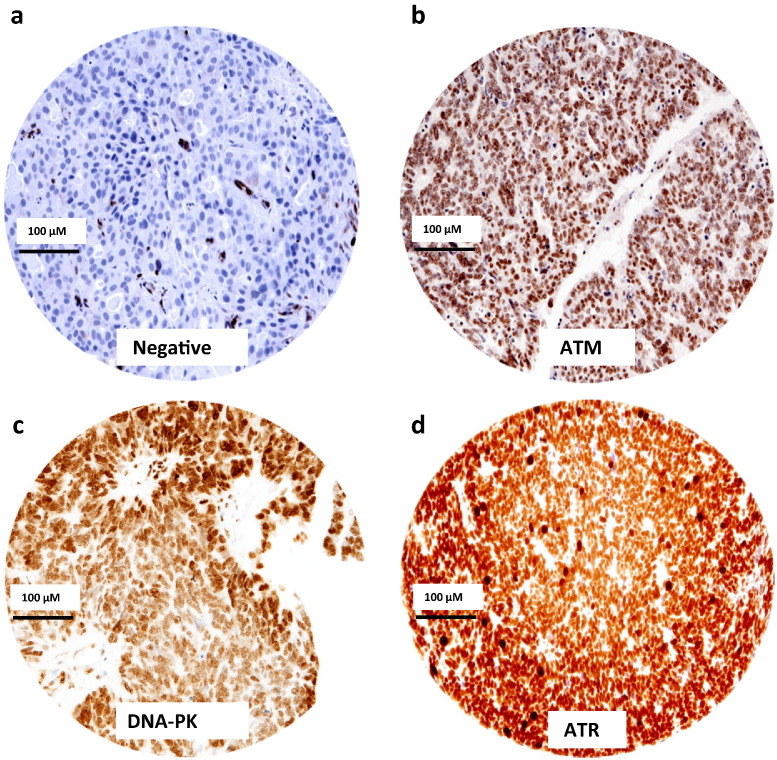
Microphotographs of ATM, DNA-PKcs and ATR expressions in ovarian epithelial cancer tissue (scale bar, 100 μM). (**a**) Negative, (**b**) high ATM protein expression, (**c**) high DNA-PK protein expression, (**d**) high ATR protein expression.

A total of 186 tumours were suitable for analysis of ATM nuclear expression. 135/186 (72.6%) tumours were low for ATM expression and 51/186 (27.4%) of the tumours were high for ATM expression (Figs. 1a and b). High ATM expression was significantly associated with serous cystadenocarcinomas (p = 0.021), CA-125 response to chemotherapy (p = 0.017) and platinum resistance (p = 0.017). High ATM is significantly associated with high XRCC1 (p = 0.002). High ATM is also significantly associated with biomarkers involved in cell cycle regulation such as high CDC45 (p = 0.027), high CDK1 (p = 0.001), and Ki67 (p = 0.001) (Table 1).

#### 3.1.2 DNA-PKcs

A total of 190 tumours were suitable for analysis of DNA-PKcs nuclear expression. 56/190 (29.4%) tumours were low for DNA-PKcs nuclear expression and 134/190 (70.5%) of the tumours were high for DNA-PKcs nuclear expression (Fig. 1c). High DNA-PKcs expression was significantly associated with serous cystadenocarcinomas (p = 0.006), FIGO stage (p = 0.018) and grade (p = 0.004). High DNA-PKcs is significantly associated with high XRCC1 (p = 0.009). High DNA-PKcs is also significantly associated with biomarkers involved in cell cycle regulation such as high CDC45 (p = 0.001) and high CDK1 (p = 0.001) (Table 2).

#### 3.1.3 ATR

A total of 177 tumours were suitable for analysis of ATR expression. 120/177 (67.8%) tumours was low for ATR nuclear expression and 57/177 (32.2%) of the tumours was high for ATR nuclear expression (Fig. 1d). There were no significant associations between ATR expression, clinicopathological variables or XRCC1 (Supplementary Table S3).

### 3.2 Survival analysis

#### 3.2.1 Univariate analysis

**Fig. 2:**
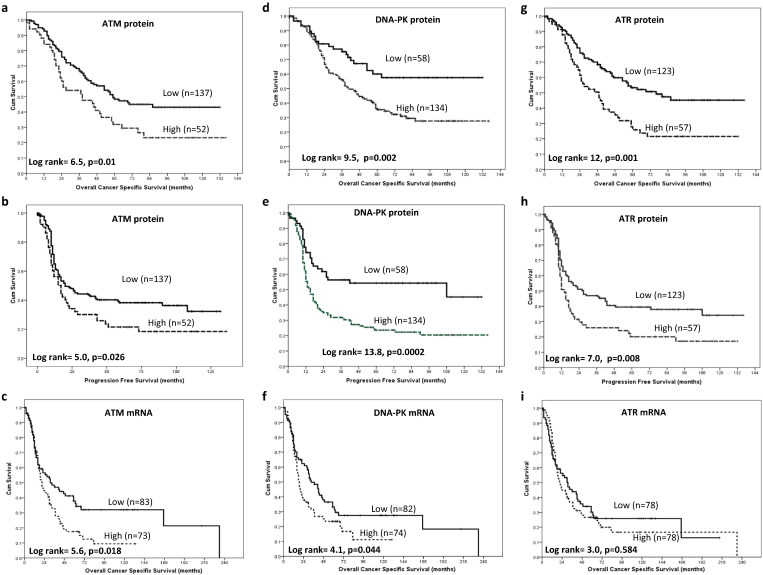
Kaplan Meier curves for ATM protein expression showing overall ovarian cancer specific survival (OVCC) (**a**), progression free survival (**b**), ATM mRNA expression and OVCC (**c**). Kaplan Meier curves for DNA-PKcs expression showing overall ovarian cancer specific survival (**d**), progression free survival (**e**) and DNA-PKcs mRNA expression and OVCC (**f**). Kaplan Meier curves for ATR expression showing overall ovarian cancer specific survival (**g**), progression free survival (**h**) and ATR mRNA expression and OVCC (**i**).

High ATM nuclear expression in tumours showed an adverse clinical outcome with poor ovarian cancer specific survival (p = 0.01) (Fig. 2a) and progression free survival (p = 0.026) (Fig. 2b) compared with tumours that had low ATM expression. At the gene expression level, high ATM mRNA expressers have poor survival compared to low ATM mRNA expressers (p = 0.018) (Fig. 2c).

High DNA-PKcs expression in tumours showed an adverse clinical outcome with poor ovarian cancer specific survival (p = 0.002) (Fig. 2d) and progression free survival (p = 0.0002) (Fig. 2e) compared with tumours that had low DNA-PKcs expression. At the gene expression level, high DNA-PKcs mRNA expressers have poor survival compared to low DNA-PKcs mRNA expressers (p = 0.0044) (Fig. 2f).

High ATR expression in tumours showed an adverse clinical outcome with poor ovarian cancer specific survival (p = 0.001) (Fig. 2g) and progression free survival (p = 0.008) (Fig. 2h) compared with tumours that had low ATR expression. At the gene expression level there was no significant difference between high and low ATM mRNA expressers (p = 0.584) (Fig. 2i).

**Fig. 3:**
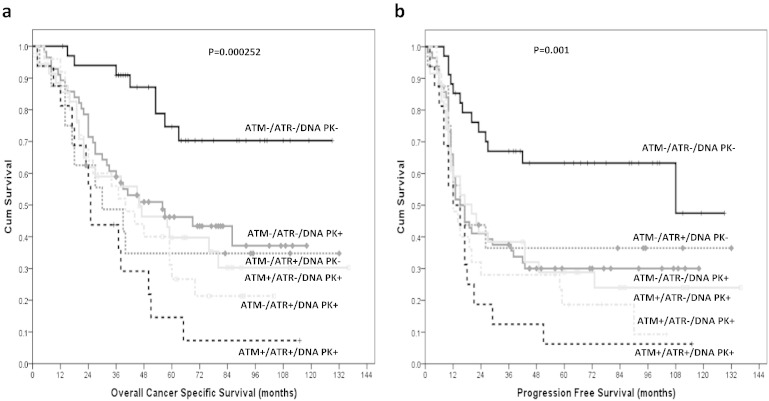
Kaplan Meier curves for ATM/ATR/DNA-PKcs expression together in ovarian tumours showing overall ovarian cancer specific survival (**a**) and progression free survival (**b**).

Investigating ATM/DNA-PKcs/ATR protein together we found that tumours that have high ATM/DNA-PKcs/ATR protein expression have poor ovarian cancer specific survival (p = 0.000252) (Fig. 3a) and progression free survival (p = 0.001) (Fig. 3b) compared to tumours that are low ATM/DNA-PKcs/ATR protein expressers.

**Fig. 4:**
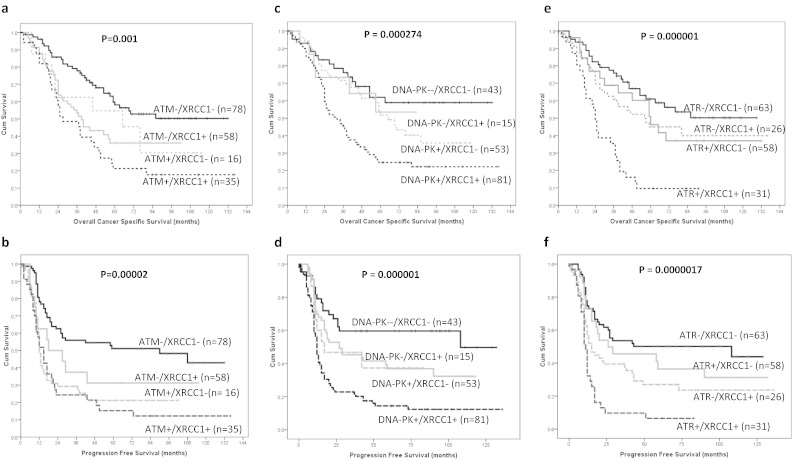
Kaplan Meier curves for ATM/XRCC1 expression together in ovarian tumours showing overall ovarian cancer specific survival (**a**), progression free survival (**b**). Kaplan Meier curves for DNA-PKcs/XRCC1 expression together in ovarian tumours showing overall ovarian cancer specific survival (**c**), progression free survival (**d**). Kaplan Meier curves for ATR/XRCC1 expression together in ovarian tumours showing overall ovarian cancer specific survival (**e**), progression free survival (**f**).

Previously we have demonstrated that XRCC1, a key player in DNA base excision repair, is an important prognostic and predictive biomarker in ovarian cancer [23]. Here, we have conducted an exploratory stratification analysis based on XRCC1 status and ATM/DNA-PKcs/ATR expression in ovarian cancer. As shown in Fig. 4a–f, ATM +/XRCC1 +, DNA-PKcs +/XRCC1 + and ATR +/XRCC1 + tumours have significantly worse ovarian cancer specific survival and progression free survival compared to tumours that are ATM-/XRCC1-, DNA-PKcs-/XRCC1- and ATR-/XRCC1- respectively (ps ≤ 0.001).

#### 3.2.2 Multivariate analysis

ATM, ATR, DNA-PKcs and XRCC1 protein expressions were investigated in a Cox multivariate model that was also adjusted for FIGO stage, grade, and residual tumour burden after surgery, chemotherapy and CA-125 response (Table 3). For ovarian cancer specific survival (OCSS), high DNA-PKcs (p = 0.043) and high ATR expression (p = 0.006) were independently associated with poor OCSS. FIGO stage (p = 0.025), residual tumour burden after surgery (p = 0.015), grade (p = 0.002) and CA-125 response (p < 0.0001) were additional factors independently associated with poor OCSS. For progression free survival (PFS), DNA-PKcs expression (p = 0.003) and XRCC1 expression (p = 0.004) were independently associated with poor PFS. FIGO stage (p < 0.0001), grade (p = 0.024), residual tumour burden after surgery (p = 0.002) and CA-125 response (p < 0.0001) were additional factors independently associated with poor PFS (Supplementary Table S4).

## 4 Discussion

Overall prognosis for advanced ovarian cancer remains poor. Resistance to platinum based chemotherapy adversely impacts patient outcome [4], [5]. DNA damage induced by platinum chemotherapy is, to a large extent, processed by the DNA damage signalling and repair machinery in cells. Up-regulation of DNA damage signalling and repair pathways may be an important cause of therapeutic resistance in ovarian cancer. ATM, DNA-PKcs and ATR are key proteins involved in DNA repair in response to DNA damaging chemotherapy [6], [7], [8], [9], [10], [11], [12], [13], [14]. Altered expression of ATM, DNA-PKcs and ATR may have prognostic and predictive significances in ovarian cancer.

In the current study we have provided evidence that ATM, DNA-PKcs and ATR are promising biomarkers in ovarian cancer. We found that high ATM expression was associated with serous cystadenocarcinomas, poor response to chemotherapy and platinum resistance. High DNA-PKcs expression was associated with serous cystadenocarcinomas, advanced stage and high grade tumours. In univariate analysis, high ATM, high DNA-PKcs and high ATR expression are associated with poor ovarian cancer specific survival (OCSS) and progression free survival (PFS). Taken together, ATM +/DNA-PKcs +/ATR + tumours had the worst survival compared to ATM-/DNA-PK-/ATR- tumours. In a separate cohort, the adverse prognostic significance was also observed at the mRNA level for ATM and DNA-PKcs implying that high protein levels may be related to high ATM and DNA-PKcs mRNA levels in tumours. For ATR, mRNA levels were not significant implying that post-transcriptional mechanisms may be operating in certain tumours to increase ATR protein levels. In fact, pre-clinical evidence that such a mechanism may be operating to control ATR protein levels has recently been demonstrated [24]. A limitation in the current study is that we were unable to compare protein and mRNA expressions in the same cohort. Nevertheless, the clinical data presented here does suggest that high ATM/DNA-PKcs/ATR expressing tumours may be less sensitive to chemotherapy. The data is entirely consistent with pre-clinical studies demonstrating an essential role for ATM, DNA-PKcs and ATR in determining platinum sensitivity in cancer cell line models. Depletion or inhibition by small molecule inhibitors has been shown to result in platinum sensitivity [25], [26], [27], [28], [29]. In multivariate Cox model for OCSS, high DNA-PKcs and high ATR expression was independently associated with poor survival providing further evidence for prognostic and predictive significance in ovarian cancer. However, as discussed previously, the data is retrospective and is in need of prospective validation in larger multicentre studies. Another limitation to the study is that we have investigated ATM, DNA-PKcs and ATR expressions only in static states. Moreover, post-translational modification of downstream proteins such as phosphorylation of Chk1 and Chk2 are essential for functional capacity of pathways. Analyses of expression of phosphorylated Chk1, phosphorylated Chk2 and autophosphorylated forms for ATM, DNA-PKcs and ATR may provide further insights into the clinicopathological significance of the DNA damage signalling pathways in ovarian cancers.

Recent studies have demonstrated significant cross-talk between XRCC1 (a key player in base excision repair (BER) and single strand break repair) and DDR [30], [31], [32]. ATM and DNA-PKcs are known to be involved in the phosphorylation of XRCC1 to promote BER [30], [31]. We have recently shown that XRCC1 is key predictive biomarker of platinum resistance in ovarian cancers [23]. In the current study we therefore explored if patient stratification could be achieved based on XRCC1, ATM, DNA-PKcs and ATR expression statuses in tumours. ATM +/XRCC1 +, DNA-PK +/XRCC1 + and ATR +/XRCC1 + tumours had the worst survival compared to ATM-/XRCC1-, DNA-PK-/XRCC1- and ATR-/XRCC1- tumours in our study. However, a limitation of our study is that it is retrospective and involves a limited number of patients. Larger studies are required to confirm our findings. The recent success of PARP1 inhibitors (that block BER and SSBR) in germ-line BRCA deficient ovarian cancer [33], [34] provides evidence that targeting DNA repair is an important area for personalization of ovarian cancer therapy. Although the data in germ-line BRCA deficient tumours is promising, the search for such synthetic lethal relationships in the more common sporadic epithelial ovarian cancer remains an area of on-going investigation. In a recent pre-clinical study, we have demonstrated that ATM, DNA-PKcs and ATR inhibitors are synthetically lethal in XRCC1 deficient cancer cells [35], [36]. Taken together, our data suggests that XRCC1 based personalization using ATM, DNA-PKcs or ATR inhibitors may be feasible and this approach clearly warrants further investigation in vivo in sporadic epithelial ovarian cancers. The associations demonstrated herein between ATM, DNA-PKcs and cell cycle markers such as CDK1 and CDC25 are also consistent with the known roles of ATM and DNA-PKcs during cell cycle progression [37].

In conclusion, we have provided evidence that ATM, DNA-PKcs and ATR are promising prognostic and predictive biomarkers in ovarian cancer. Our data supports a rational approach using small molecule inhibitors of ATM, DNA-PKcs and ATR, currently under pharmaceutical development, for ovarian cancer therapy.

## Conflicts of interest

None.

The following are the supplementary data related to this article.

Supplementary material.

Supplementary Fig. S1 Negative controls with no primary antibody were included in each run and shown here.

Supplementary tables.

## Figures and Tables

**Table 1:** ATM expression and epithelial ovarian cancer.

Markers		ATM (Low)	ATM (high)	p Value
Pathological parameters		Number (%)	Number (%)
Tumour type	Serous	71 (52.6)	37 (72.5)	**0**.**021**
Mucinous	10 (7.4)	4 (7.8)
Endometroid	31 (23.0)	6 (11.8)
Clear cell	22 (16.3)	2 (3.9)
Others	1 (0.7)	2 (3.9)
FIGO stage	I	47 (34.3)	12 (23.5)	0.470
II	19 (13.9)	8 (15.7)
III	59 (43.1)	24 (47.1)
IV	12 (8.8)	7 (13.7)
Grade	1	16 (12.4)	5 (9.8)	0.524
2	17 (13.2)	10 (19.6)
3	96 (74.4)	36 (70.6)
CA125 response	CR	120 (88.9)	35 (85.2)	**0**.**017**
None CR	15 (11.1)	12 (14.8)
Platinum sensitivity	Sensitive	95 (71.4)	31 (63.3)	**0**.**017**
Resistant	38 (28.6)	18 (36.7)
XRCC1	Low	78 (57.4)	16 (31.4)	**0**.**002**
High	58 (42.6)	35 (68.6)
ATR	Low	90 (68.7)	33 (67.3)	0.862
High	41 (31.3)	16 (32.7)
DNA-PK	Low	45 (36.3)	1 (2.3)	**1**.**7 × 10**^− **5**^
High	79 (63.7)	42 (97.7)
CDC45	Low	22 (19.1)	2 (4.8)	**0**.**027**
High	93 (80.9)	40 (95.2)
CDK1	Low	38 (34.9)	2 (5.4)	**0**.**001**
High	71 (65.1)	35 (94.6)
Ki67	Low	32 (51.6)	6 (14.6)	**0**.**001**
High	30 (48.4)	35 (85.4)

Significant p Values are in bold.

**Table 2:** DNA-PK and epithelial ovarian cancer.

Markers		DNA-PK (Low)	DNA-PK (High)	p Value
Pathological parameters		Number (%)	Number (%)
Tumour type	Serous	23 (41.1%)	88 (65.7%)	**0**.**006**
Mucinous	6 (10.7%)	8 (6.0%)
Endometroid	14 (25.0%)	24 (17.9%)
Clear cell	13 (23.2%)	11 (8.2%)
Others	0 (0%)	3 (2.2%)
FIGO stage	I	26 (46.4%)	33 (24.3%)	**0**.**018**
II	7 (12.5%)	20 (14.7%)
III	20 (35.7%)	65 (47.8%)
IV	3 (5.4%)	18 (13.2%)
Grade	1	13 (24.5%)	9 (6.9%)	**0.004**
2	6 (11.3%)	21 (16.0%)
3	34 (64.2%)	101 (77.1%)
CA125 response	CR	40 (90.9)	95 (81.2)	0.136
None CR	4 (9.1)	22 (18.8)
Platinum sensitivity	Sensitive	34 (77.3)	74 (63.2)	0.091
Resistant	10 (22.7)	43 (36.8)
XRCC1	Low	30 (66.7)	53 (43.8)	**0**.**009**
High	15 (33.3)	68 (56.2)
ATM	Low	45 (97.8)	79 (65.3)	**0**.**001**
High	1 (2.2)	42 (34.7)
ATR	Low	31 (67.4)	81 (66.9)	0.956
High	15 (32.6)	40 (33.1)
CDC45	Low	13 (28.9)	9 (8.6)	**0**.**001**
High	32 (71.1)	96 (91.4)
CDK1	Low	25 (59.5)	12 (12.2)	**0**.**001**
High	17 (40.5)	86 (87.8)
Ki67	Low	35 (76.1)	76 (63.3)	0.118
High	11 (23.9)	44 (36.7)

Significant p Values are in bold.

**Table 3:** Multivariate analysis.

	p Value	Exp (B)	95% CI for Exp (B) Lower upper	
*Cancer specific survival*				
ATM	0.933	1.007	0.862	1.176
ATR	**0**.**006**	1.218	1.058	1.401
DNA-PK	**0**.**043**	1.389	1.010	1.911
XRCC1	0.111	1.195	0.960	1.487
Ca125 response	**1**.**4** × **10**^− **4**^	1.656	1.278	2.147
Chemotherapy regimen	0.659	1.115	0.687	1.811
FIGO stage	**0**.**025**	1.420	1.046	1.928
Grade	**0**.**002**	2.029	1.300	3.166
Residual burden	**0**.**015**	1.548	1.088	2.201

*Progression free survival*				
ATM	0.261	0.919	0.793	1.065
ATR	0.225	1.086	0.950	1.240
DNA-PK	**0**.**003**	1.550	1.166	2.060
XRCC1	**0**.**004**	1.353	1.101	1.663
Ca125 response	**1**.**9** × **10**^− **9**^	2.230	1.716	2.898
Chemotherapy regime	0.200	1.323	0.863	2.028
FIGO stage	**1**.**6** × **10**^− **5**^	**1**.**760**	1.334	2.322
Grade	**0**.**024**	**1**.**558**	1.059	2.293
Residual burden	**0**.**002**	1.612	1.194	2.177

Significant p Values are in bold.
