# Supplementary material for: ATM, ATR and DNA-PKcs expressions correlate to adverse clinical outcomes in epithelial ovarian cancers
Source: BBA Clin. 2014 Aug 14;2:10–7. doi: 10.1016/j.bbacli.2014.08.001 (PMC4633921; doi:10.1016/j.bbacli.2014.08.001)
Supplement: Supplementary file 1 — Supplementary material. [file mmc1.docx]

**Optimization of antibodies for ATM, ATR and DNA-PKcs (supplementary data 1)**

**ATM:** ATM antibody dilutions were performed at 1/50, 1/100, 1/150 and 1/200 as per manufacturer’s recommendation with incubation times of 18 hours. Concentration that generated tumour specific staining with no background staining was chosen for further IHC.

**ATR:** ATR antibody dilutions were performed at 1/10, 1/20, 1/30, 1/40, 1/100, 1/125, 1/150, 1/175 and 1/200. Incubation times of 30 minutes or 60 minutes were investigated.

**DNA-PKcs:** DNA-PKcs, antibody dilutions were performed at 1/200, 1/400, 1/600, 1/800, 1/900, 1/1000 and 1/1100.

The optimal dilutions were as follows: ATM (1:100, 18 hours incubation), ATR (1:20

18 hours incubation), DNA-PKcs (1:1000, 60 min incubation). Negative controls with no primary antibody were included in each run and shown in supplementary figure S1.
