# Supplementary material for: ATM, ATR and DNA-PKcs expressions correlate to adverse clinical outcomes in epithelial ovarian cancers
Source: BBA Clin. 2014 Aug 14;2:10–7. doi: 10.1016/j.bbacli.2014.08.001 (PMC4633921; doi:10.1016/j.bbacli.2014.08.001)
Supplement: Supplementary Fig. S1 — Negative controls with no primary antibody were included in each run and shown here. [file mmc2.pptx]

## Slide 1
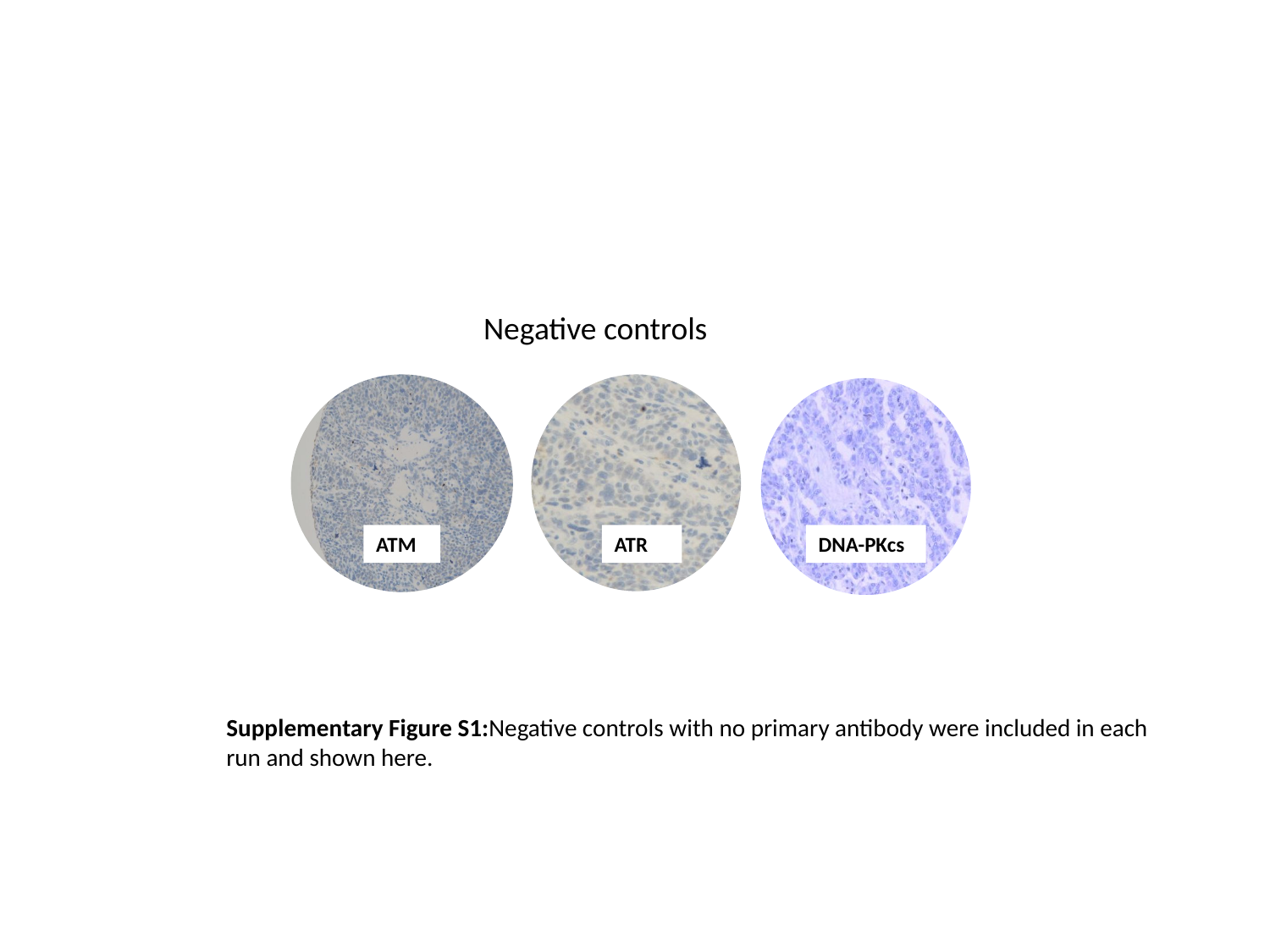

Negative controls
ATM
ATR
DNA-PKcs
Supplementary Figure S1:Negative controls with no primary antibody were included in each run and shown here.
