# Supplementary material for: ATM, ATR and DNA-PKcs expressions correlate to adverse clinical outcomes in epithelial ovarian cancers
Source: BBA Clin. 2014 Aug 14;2:10–7. doi: 10.1016/j.bbacli.2014.08.001 (PMC4633921; doi:10.1016/j.bbacli.2014.08.001)
Supplement: Supplementary file 3 — Supplementary tables. [file mmc3.docx]

**Supplemetary Table S1.** Patient demographics and pathological features in ovarian cancer.

|  | | **Number** | | **Percentages** |
| --- | --- | --- | --- | --- |
| **Characteristics** | |  |  |  |
| ***Pathology*** | | | | |
|  | Serous cystadenocarcinoma |  | 111 | 58.4% |
|  | Endometrioid |  | 38 | 20.0% |
|  | Clear cell carcinoma |  | 24 | 12.6% |
|  | Mucinous cystadenocarcinoma |  | 14 | 7.4% |
|  | Others |  | 7 | 3.6% |
| ***Grade*** | | | | |
|  | 1 |  | 22 | 11% |
|  | 2 |  | 28 | 15% |
|  | 3 |  | 143 | 74% |
| ***Residual tumour*** | | | | |
|  | None/Microscopic |  | 109 | 57% |
|  | <1cm |  | 14 | 7 |
|  | >1-2 cm |  | 17 | 9 |
|  | >2cm |  | 53 | 27% |
| ***FIGO Stage*** | | | | |
|  | IC |  | 58 | 30% |
|  | II |  | 27 | 14% |
|  | III |  | 85 | 43% |
|  | IV |  | 25 | 13% |
| ***Chemotherapy*** | | | | |
|  | Carboplatin monotherapy |  | 83 | 42.7% |
|  | Carboplatin + Paclitaxel |  | 111 | 49.5% |
| ***Platinum sensitivity*** | | | | |
|  | Sensitive |  | 126 | 65% |
|  | Resistant |  | 60 | 31% |
|  | Unknown |  | 9 | 4% |
| ***Relapse status*** | | | | |
|  | Progression-free |  | 64 | 39% |
|  | Progressed/relapsed |  | 130 | 61% |
| ***Survival status*** | | | | |
|  | Living |  | 83 | 42.8% |
|  | Dead |  | 111 | 57.2% |

**Supplementary Table S2:** Antigens, primary antibodies, clone, source, optimal dilution and scoring system used for each immunohistochemical marker

| **Antigen** | **Antibody** | **Clone** | **Source** | **Antigen Retrieval** | **Dilution / Incubation Time** | **Distribution** | **Scoring**  **system** | **Cut-offs** |
| --- | --- | --- | --- | --- | --- | --- | --- | --- |
| ATM | Rabbit MAb anti-ATM | Y170 | Abcam | Citrate pH6  95^0^C for 20 min | 1:100  18 hours | Nuclear | % of positive cells | <25% (negative) |
| ATR | Mouse MAb anti-ATR | 1E9 | Novus Biologicals | Citrate pH6  95^0^C for 20 min | 1:20  18 hours | Nuclear | % of positive cells | >10% (positive) |
| DNA-PK | Mouse MAb anti- DNA-PK | 3H6 | Abcam | Citrate pH6  95^0^C for 20 min | 1:1000  60 min | Nuclear | H-score | H-score>180  (positive) |
| XRCC1 | Mouse MAb Anti-XRCC1 | 33-2-5 | Thermo-scientific | Citrate pH6  95^0^C for 20 min | 1:200  20 min | Nuclear | % of positive cells | ≥10% (positive) |
| Ki-67 | Mouse MAb anti-Ki-67 | MIB1 | Dako-Cytomation | Citrate pH6  95^0^C for 20 min | 1:300  60 min | Nuclear | % of positive cells | 0-30% (low)  >30% (high) |
| CDC45L | Rabbit MAb anti-CDC45L | EPR5758 | Abcam | Citrate pH6  95^0^C for 20 min | 1:100  30 min | Nuclear | % of positive cells | ≥10% (positive) |
| CDK1 | Mouse MAb anti-CDK1 | A17 | Abcam | EDTA pH8  95^0^C for 25 min, staged cool down | 1:300  30 min | Nuclear | H-score | H-score>150  (positive) |

**Supplementary Table S3.** ATR and epithelial ovarian cancer.

| **Markers** | | **ATR (Low)** | **ATR (High)** | **P value** |
| --- | --- | --- | --- | --- |
| **Pathological parameters** | | **Number (%)** | **Number (%)** |  |
| **Tumour type** | Serous | 64 (53.3) | 41 (71.9) | 0.132 |
|  | Mucinous | 8 (6.7) | 4 (7.0) |  |
|  | Endometroid | 27 (22.5) | 8 (14.0) |  |
|  | Clear cell | 18 (15.0) | 4 (7.0) |  |
|  | Others | 3 (2.5) | 0 (0.0) |  |
| **FIGO Stage** | I | 42 (34.4) | 12 (21.1) | 0.104 |
|  | II | 17 (13.9) | 8 (14.0) |  |
|  | III | 54 (44.3) | 27 (47.4) |  |
|  | IV | 9 (7.4) | 10 (17.5) |  |
| **Grade** | 1 | 14 (12.2) | 5 (8.9) | 0.617 |
|  | 2 | 15 (13.0) | 10 (17.9) |  |
|  | 3 | 86 (74.8) | 41 (73.2) |  |
| **CA125 Response** | CR | 102 (87.2)) | 44 (78.6) | 0.144 |
|  | None CR | 15 (12.8) | 12 (21.4) |  |
| **Platinum Sensitivity** | Sensitive | 85 (72.6) | 33 (58.9) | 0.070 |
|  | Resistant | 32 (27.4) | 23 (41.1) |  |
| **XRCC1** | Low | 63 (52.1) | 26 (45.6) | 0.422 |
|  | High | 58 (47.9) | 31 (54.4) |  |
|  | High | 60 (55.0) | 29 (58.0) |  |
|  | High | 33 (26.8) | 16 (28.1) |  |
| **DNA-PK** | Low | 31 (27.7) | 15 (27.3) | 0.956 |
|  | High | 81 (72.3) | 40 (72.7) |  |
| **CDC45** | Low | 16 (15.0) | 8 (16.0) | 0.865 |
|  | High | 91 (85.0) | 42 (84.0) |  |
| **CDK1** | Low | 29 (29.0) | 10 (22.2) | 0.394 |
|  | High | 71 (71.0) | 35 (77.8) |  |
| **Ki67** | Low | 74 (64.9) | 41 (73.2) | 0.277 |
|  | High | 40 (35.1) | 15 (26.8) |  |
